# Gene expression analyses in maize inbreds and hybrids with varying levels of heterosis

**DOI:** 10.1186/1471-2229-8-33

**Published:** 2008-04-10

**Authors:** Robert M Stupar, Jack M Gardiner, Aaron G Oldre, William J Haun, Vicki L Chandler, Nathan M Springer

**Affiliations:** 1 Center for Plant and Microbial Genomics, Department of Plant Biology, University of Minnesota, Saint Paul MN 55108, USA; 2 Department of Plant Science, and BIO5 Institute, University of Arizona, Tucson, AZ 85721, USA; 3 Department of Agronomy and Plant Genetics, University of Minnesota, Saint Paul MN 55108, USA

## Abstract

**Background:**

Heterosis is the superior performance of F_1 _hybrid progeny relative to the parental phenotypes. Maize exhibits heterosis for a wide range of traits, however the magnitude of heterosis is highly variable depending on the choice of parents and the trait(s) measured. We have used expression profiling to determine whether the level, or types, of non-additive gene expression vary in maize hybrids with different levels of genetic diversity or heterosis.

**Results:**

We observed that the distributions of better parent heterosis among a series of 25 maize hybrids generally do not exhibit significant correlations between different traits. Expression profiling analyses for six of these hybrids, chosen to represent diversity in genotypes and heterosis responses, revealed a correlation between genetic diversity and transcriptional variation. The majority of differentially expressed genes in each of the six different hybrids exhibited additive expression patterns, and ~25% exhibited statistically significant non-additive expression profiles. Among the non-additive profiles, ~80% exhibited hybrid expression levels between the parental levels, ~20% exhibited hybrid expression levels at the parental levels and ~1% exhibited hybrid levels outside the parental range.

**Conclusion:**

We have found that maize inbred genetic diversity is correlated with transcriptional variation. However, sampling of seedling tissues indicated that the frequencies of additive and non-additive expression patterns are very similar across a range of hybrid lines. These findings suggest that heterosis is probably not a consequence of higher levels of additive or non-additive expression, but may be related to transcriptional variation between parents. The lack of correlation between better parent heterosis levels for different traits suggests that transcriptional diversity at specific sets of genes may influence heterosis for different traits.

## Background

Heterosis is the phenomenon in which F_1 _hybrids exhibit phenotypes that are superior to their parents [1,2]. Plant breeders have utilized heterosis for the development of superior yielding varieties in many important crop species without fully understanding the molecular basis of heterosis. Researchers frequently discuss the magnitude of yield heterosis for a particular hybrid. In maize, the different inbred lines have been divided into "heterotic groups" based upon the level of grain yield heterosis [3]. Generally, crosses within heterotic groups have lower grain yield heterosis than crosses between groups. However, heterotic groups are used as a general tool and not as a precise predictor of heterotic response [4]. There is a correlation between grain yield heterosis and genetic diversity such that increasing genetic diversity produces increasing level of grain yield heterosis [5]. However, when the parents become highly diverse this relationship is no longer observed [3,6].

Although heterosis in crop plants is most commonly discussed in terms of yield, numerous other phenotypic traits also display heterosis. Maize exhibits high levels of heterosis for many traits such as root growth, height, ear node, leaf width, seedling biomass and other traits [7-11]. Within a given hybrid, the amount of heterosis can vary widely for different traits [9,12].

**Figure 1:**
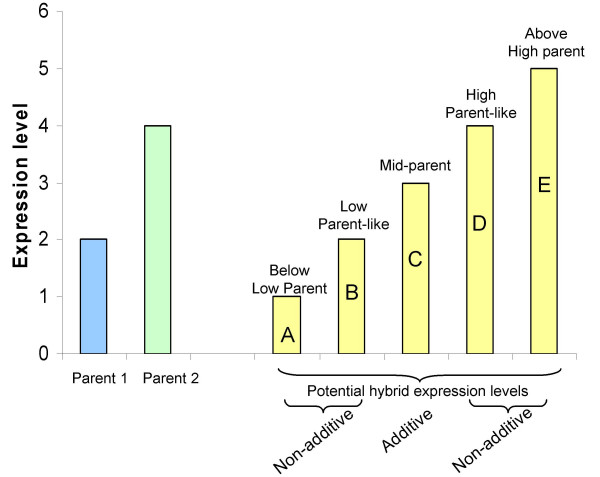
**Schematic diagram of potential patterns of hybrid gene expression**. This hypothetical gene exhibits higher expression in parent 2 than in parent 1. Five different potential patterns of hybrid expression (A-E) are diagrammed. The hybrid could exhibit (A) below-low parent expression (BLP); (B) low parent-like expression (LP); (C) mid-parent expression; (D) high parent-like expression (HP); or (E) above high parent expression (AHP). Only mid-parent expression is classified as additive. The BLP, LP-like, HP-like and AHP expression patterns would all be examples of non-additive expression.

While it is widely agreed that parental genetic diversity serves as the basis of heterosis, the specific aspects of genetic diversity and how these contribute to heterotic phenotypes remains to be determined. The molecular mechanism(s) driving heterotic phenotypes remains a subject of wide interest and debate [12,13]. The availability of high-throughput gene expression profiling technologies has allowed researchers to study the gene expression profile of hybrid plants relative to the inbred parents [11,14-21]. In general, most of these studies have focused on characterizing gene expression patterns in a single heterotic hybrid compared to the two parents. Many of these studies have addressed similar topics regarding gene expression and heterosis, such as the relative frequencies of additive and non-additive expression levels in the hybrid. Additive expression occurs when the hybrid expression level is equivalent to the mid-parent values while non-additive expression occurs whenever the hybrid expression level deviates from the mid-parent level (Figure 1). It is worth noting that non-additive expression phenotypes can include expression levels between the mid-parent and parental values, expression levels equivalent to one of the parents or expression levels outside the parental range. The identity and frequency of genes exhibiting hybrid gene expression levels outside of the parental range have been of particular interest in these studies.

The hybrid expression profiling studies have utilized a variety of expression profiling platforms, experimental designs and tissues. Several studies have found that the majority (~75%) of genes exhibit additive expression in the hybrid and that only small numbers of the non-additively expressed genes exhibit expression levels outside the parental range [11,15,17]. Other studies have found much higher levels of non-additive expression and numerous examples of expression outside the parental range [21-23]. It is unclear whether these differences are caused by biological differences between tissues, genotypes, or differences in the expression profiling platforms.

In this study we have investigated the heterosis and gene expression profiles for a set of maize hybrids with varying levels of parental genetic diversity. In addition, gene expression profiling was performed using several different technologies enabling the assessment of whether hybrids that generally exhibit lower levels of heterosis exhibit lower levels of non-additive expression or expression levels outside the parental range.

## Results

### Different maize hybrids show a range of heterotic responses that vary among traits

The primary objective of this study was to identify, and compare levels of, non-additive gene expression in several maize hybrids with varying levels of heterosis. There is a substantial amount of prior research on the levels of heterosis for grain yield in various maize hybrids. However, our expression profiling was performed with seedling tissue and this tissue may not be directly related to grain yield phenotypes. Therefore, we monitored maize inbreds and hybrids to assess the levels of better parent heterosis (BPH) for five different phenotypes, including two different seedling phenotypes. BPH is represented as the percent phenotypic increase in the hybrid relative to the better parent phenotype (see Methods for BPH equation). Our goal was to identify whether the levels of heterosis for different hybrid genotypes were correlated among a variety of traits, thus allowing us to determine which hybrids exhibit higher or lower "overall" heterosis.

**Figure 2:**
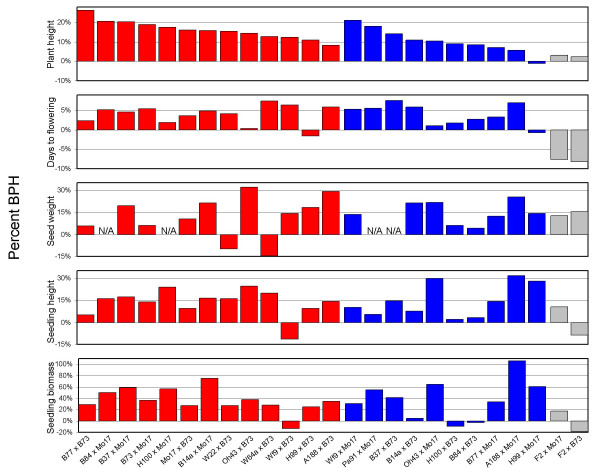
**Heterosis for non-yield traits**. The percent BPH is shown for all traits and all hybrids scored in this study. The numerical BPH values are available in Additional file 2. Red bars represent BPH for hybrids generated between SS and NSS inbreds, blue bars represent BPH for hybrids generated within SS and NSS inbreds, and grey bars represent BPH for hybrids derived from an inbred line with mixed origin (F2).

We measured the mature plant height, 50-seed weight, days to flowering, seedling plant height and seedling biomass BPH levels for a series of hybrids. The inbred lines B73 or Mo17 were used as paternal parents in all hybrids studied. The phenotypic values for each replicate of all five traits are provided in Additional file 1 and the BPH values are available in Figure 1 and Additional file 2. The relative BPH levels were quite variable among the different traits (Figure 2). For example, Oh43 × B73 exhibited the highest BPH for seed weight, but the fifth lowest BPH for days to flowering (Figure 2; see Additional file 2). We tested whether there was a correlation in the level of BPH among hybrids for any two traits [see Additional file 3]. Seedling height and seedling biomass exhibited a strong correlation (*p *< 0.0001) while plant height and days to flowering exhibited a weaker, but significant, correlation (*p *= 0.013). The other eight trait comparisons did not show significant correlations. Thus, in general, the level of BPH heterosis for one trait is a poor predictor of the level of heterosis for another trait.

**Figure 3:**
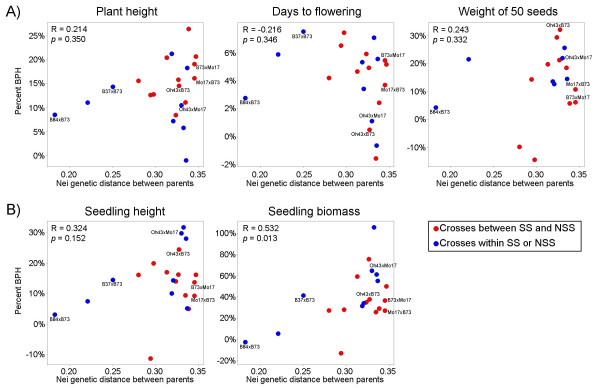
**Relationship between parental genetic diversity and hybrid heterosis among traits and hybrids**. The percentage better parent heterosis (BPH) for each hybrid is plotted against the genetic distance between parents. The 25 hybrids were scored based on percentage BPH for five traits (plant final height, days to flowering, weight of 50 seeds, 11-day height and 11-day biomass). Traits measured on field-grown plants are shown in (A) and traits measured on greenhouse-grown plants are shown in (B). Average percent BPH is shown based on two field replicates (A) and three greenhouse replicates (B). Spots representing crosses between stiff stalk (SS) and non-stiff stalk (NSS) groups are shown in red, and spots representing crosses within either group are shown in blue. The Pearson's R correlation value and *p*-value of the regression are shown for each trait. The six hybrids that were used for expression profiling are labelled in each of the five plots.

We assessed whether the concept of heterotic groups, which was developed as a tool to enable breeding for grain yield [4], would predict heterosis levels for other traits. The concept of heterotic groups predicts that crosses within a heterotic group will generally exhibit less heterosis than crosses between heterotic groups. For all five traits we monitored, there were multiple intra-heterotic group crosses that exhibited higher levels of heterosis than several of the inter-heterotic group hybrids. For example, while B37 × B73 is an intra-heterotic group cross it displayed heterosis levels among traits that were similar to, and sometimes superior to, inter-heterotic group hybrids made between more distant parental genotypes (Figure 2, 3). It is worth noting that heterotic groups are not entirely defined based upon heterosis but are often influenced by relatedness and other factors [4].

We investigated the correlation between the levels of BPH and the genetic distance (based on Nei SNP genetic distances calculated by Hamblin et al. [24]) between the parent lines for each of the five traits. Four out of the five traits exhibited positive correlation values, however only seedling biomass was statistically significant (*p *= 0.013). The days to flowering phenotype exhibited a non-significant negative correlation. The hybrid line with the lowest parental genetic diversity, B84 × B73, consistently exhibited low levels of relative BPH (Figure 3). However, the lines with moderate to high levels of parental genetic diversity did not consistently show a strong correlation between heterosis levels and genetic distance.

A set of six hybrid genotypes were used for gene expression profiling. These hybrids represent intra- and inter-heterotic group crosses with a range of low to high genetic diversity between the parents and exhibit a substantial range of BPH phenotypes (the data points for these six hybrids are labelled in Figure 3). Hybrids B84 × B73 and B37 × B73 represent crosses made between members of the Stiff Stalk Synthetic heterotic group and the Oh43 × Mo17 hybrid is a cross between non-Stiff Stalk inbred lines. The other three crosses (Oh43 × B73, B73 × Mo17 and Mo17 × B73) represent hybrids derived by crossing parents from the two heterotic groups. These hybrids represent a range of genetic diversity (based on 847 SNPs measured by Hamblin et al. [24]). The B84-B73 parents have a relatively low level of genetic diversity while the B37-B73 parents encompass a moderate level of genetic diversity. The other hybrids, B73-Mo17, Oh43-B73 and Oh43-Mo17, all have higher levels of genetic diversity [24] [see Additional file 2].

### Identification of differentially expressed genes

Total RNA was isolated from above ground 11-day seedling tissues for hybrids B84 × B73, B37 × B73, Oh43 × B73, Oh43 × Mo17 and their respective inbred parental lines. RNA samples were collected for three biological replications and were processed for microarray analyses using the Affymetrix maize 18 K GeneChip platform. The 18 K maize Affymetrix array contains 17,622 probe sets that are designed to detect the expression of 13,495 genes. Some genes are represented by multiple probes sets designed to detect sense and anti-sense expression or the expression of alternative transcripts. Previously obtained Affymetrix microarray data for 11-day seedlings from genotypes B73, Mo17, B73 × Mo17 and Mo17 × B73 [17] were included in downstream analyses for comparative purposes. A comparison of the expression profile of the inbred lines, B73 and Mo17, indicated that the profiles obtained in both experiments are quite comparable.

**Figure 4:**
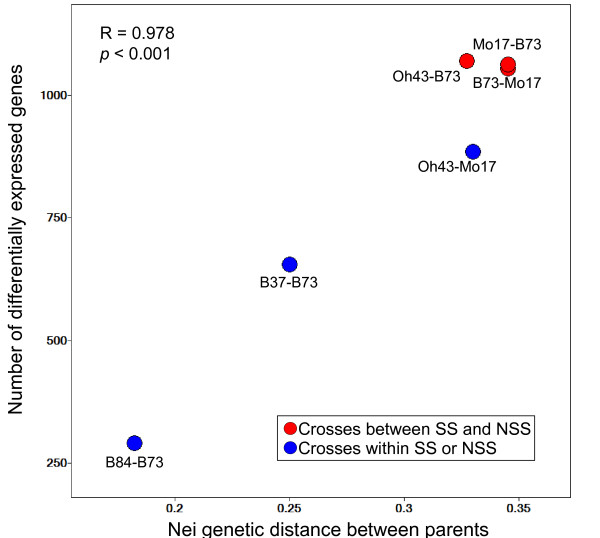
**Relationship between parental genetic diversity and differential gene expression**. The number of differentially expressed genes identified for each inbred-hybrid group based on stringent statistical criteria is plotted against the genetic distance between parents. Spots representing crosses between stiff stalk (SS) and non-stiff stalk (NSS) groups are shown in red, and spots representing crosses within either group are shown in blue. The Pearson's R correlation value and *p*-value of the regression are shown.

Genes that were differentially expressed (DE) among genotypes were identified within each inbred-hybrid group, based on an ANOVA FDR < 0.05 (and minimum signal and fold-change filters; see Methods). The numbers of DE genes were variable among the inbred-hybrid groups (Table 1). There was a strong correlation between the number of DE genes and the level of genetic distance between the parents (Figure 4). The comparison between inbred B84, inbred B73 and hybrid B84 × B73 identified 290 DE genes, by far the lowest number of any group. The comparison between inbred B37, inbred B73 and hybrid B37 × B73 identified 655 DE genes, and the remaining comparisons generated between 885–1071 DE genes (Table 1; Figure 4).

**Table 1:** Classification of differentially expressed genes based on Affymetrix microarrays

	B84 × B73	B37 × B73	Oh43 × B73	Oh43 × Mo17	Mo17 × B73	B73 × Mo17
#DE genes*	326	726	1407	993	1180	1144
# Pass filtration**	290	655	1071	885	1064	1055
#Nonadditive***	88 (30.3%)	159 (24.3%)	296 (27.6%)	233 (26.3%)	247 (23.2%)	266 (25.2%)
HP or LP****	5	32	58	47	44	55
AHP*****	0	1	3	1	0	1
BLP******	0	2	3	1	2	1

*Differentially expressed genes (based on ANOVA FDR < 0.05)

**filters: 1) at least one genotype avg signal > 50; fold-change of at least 1.2 between any two genotypes (parent1-parent2 or parent1-hybrid or parent2-hybrid comparisons)

***based on two-tailed t-test between midparent and hybrid (p < 0.05)

****based on two-tailed t-tests (p < 0.05); hybrid must be significantly different than midparent and not significantly different from either high or low parent

*****AHP: above high parent; based on one-tailed t-test between high parent and hybrid (p < 0.05) and d/a > 1

******BLP: below low parent; based on one-tailed t-test between low parent and hybrid (p < 0.05) and d/a < -1

**Figure 5:**
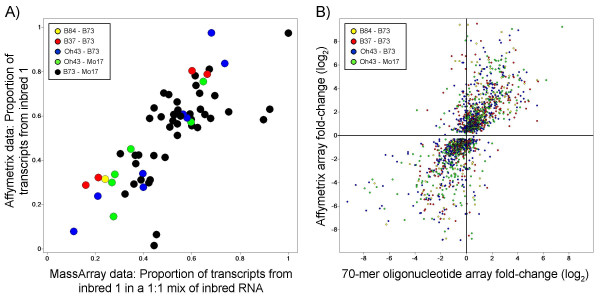
**Validation of differential expression using MassArray and 70-mer platforms**. The magnitude of differential expression between inbred lines based on the Affymetrix data was compared to the magnitude of differential expression detected using the MassArray platform and 70-mer microarray platform. The subset of the genes identified as differentially expressed on the Affymetrix platform (FDR < 0.05, and additional quality control filters; see Methods) was used for these analyses. The color coding of the data points indicates the inbred genotype comparison. (A) The same inbred RNA samples used for Affymetrix microarray analyses were mixed in a pairwise 1:1 ratio and subjected to MassArray relative allelic quantification [25]. The correlation between the MassArray proportions and the proportions calculated from the Affymetrix dataset (inbred 1 signal divided by the sum of the two inbred signals) are shown. Each spot represents the proportion of one allele per inbred-inbred comparison. The B73 and Mo17 sequence SNPs were used to design the assays, thus this comparison is most highly represented in this analysis. (B) Many genes that were determined to be differentially expressed in the Affymetrix dataset were also present on the 70-mer microarray platform. The correlation of the inbred expression fold-differences on the 70-mer oligonucleotide microarray and the Affymetrix microarray are shown. Each spot represents the fold-differences of one gene per inbred-inbred comparison. The 70-mer microarray data validated the directionality of the Affymetrix microarray patterns in 84–91% of the differentially expressed profiles (see main text).

The use of microarray expression profiling for intraspecific comparisons can be complicated by the presence of sequence polymorphisms within different inbred lines [25]. We assessed the frequency of false-positive DE genes in our Affymetrix dataset by validating the microarray data using two independent methodologies. First, the Sequenom MassArray platform was used to validate calls of differential expression between different inbred lines. We had previously used the MassArray platform to measure allele-specific expression levels for a set of ~300 genes using the same RNA samples as were used in the Affymetrix analyses [26]. The MassArray platform can detect the relative allelic proportions for a given gene in a mix of parental RNAs. The relative proportion detected for each allele can be compared with the proportion predicted based on the Affymetrix data, as was demonstrated in Stupar and Springer [17]. Fifty-six genes that were DE in the Affymetrix data were subjected to MassArray validation (this includes six genes that were DE in two different inbred-hybrid groups, resulting in validation assays for 62 DE profiles). The correlation between the Affymetrix and MassArray data was strong, with 58 of the 62 examples showing similar directionality of biased expression in both platforms (Figure 5A). A statistical analysis indicated that 74% (46/62) of the genes exhibit significant differential expression in the MassArray dataset. Second, we utilized a maize 70-mer oligonucleotide microarray platform [27] to validate the DE genes observed in the Affymetrix dataset. The same sets of RNA samples were labelled and hybridized to the 70-mer oligonucleotide microarray containing ~43,000 features. We identified a set of 13,874 features on this platform that are expected to detect the same transcripts as the Affymetrix platform. For all Affymetrix DE genes that are present on the 70-mer oligonucleotide microarray we compared the log_2 _expression differences between parental inbred lines on both platforms (Figure 5B). Pearson R values indicated significant correlations (p < 0.0001) for all of the comparisons (R = 0.697 for B84 versus B73; R = 0.679 for B37 versus B73; R = 0.720 for Oh43 versus B73; R = 0.750 for Oh43 versus Mo17). The 70-mer oligonucleotide microarray platform confirmed the directionality of the expression differences between parental inbred genotypes for the vast majority of the genes identified by Affymetrix (Figure 5B; ~91% for B84 versus B73; ~84% for B37 versus B73; ~84% for Oh43 versus B73; ~91% for Oh43 versus Mo17). While there are some examples in which differential expression is only detected using one of the platforms, the majority of genes exhibited similar differential expression in both microarray platforms. Both the Sequenom MassArray and 70-mer oligonucleotide microarray analyses indicate that the majority of the DE profiles identified using the Affymetrix microarrays were valid.

### Assessment of hybrid expression additivity

We compared the levels of additive and non-additive expression in this series of six hybrid genotypes. An initial visual assessment using clustered heat map expression profiles indicated that the six hybrids were exhibiting additive or near-additive expression levels compared to the respective parental genotypes [see Additional file 4]. To assess the proportions of statistically additive and non-additive expression patterns in the hybrids, we conducted *t*-tests of the hybrid expression values versus the inbred mid-parent values for all DE genes. A substantial proportion of the DE genes exhibited non-additive expression patterns, however, the proportions were very similar among the six different hybrids (23.2–30.3%; Table 1). No obvious trend was identified between parental genetic diversity and non-additive expression. In fact, the hybrid with the least amount of genetic diversity, B84 × B73, exhibited the greatest (30.3%) proportion of non-additive genes relative to the other hybrids.

We proceeded to assess the specific classes of non-additive expression that were exhibited in these maize hybrids. A non-additive gene could exhibit expression levels that are statistically between the mid-parent and high or low parental values (hereafter referred to as 'between parent non-additive' expression), expression levels equivalent to the high parent (HP) or low parental (LP) values, or at levels above high parent (AHP) or below low parent (BLP) (Figure 1). We assessed the number of parent-like (HP or LP), AHP and BLP hybrid expression patterns within the subset of non-additively expressed genes in each of the six hybrids (Table 1). Expression profiles were assigned to the parent-like category whenever hybrid expression levels were not significantly different from either the high or low parent (based on two-tailed *t*-tests, *P *< 0.05). Expression profiles were assigned to the AHP or BLP categories whenever hybrid expression levels were significantly above the high parent or below the low parent, respectively (one-tailed *t*-test, *P *< 0.05). The remaining genes with non-additive expression exhibited between parent non-additive expression levels. Very few genes (15 total genes among the six hybrids) were AHP or BLP using these criteria. A larger fraction of the non-additively expressed genes (18.7% among the six hybrids) exhibited parental-like expression levels. The majority (~80.1% among the six hybrids) of the non-additively expressed genes exhibited between parent non-additive expression levels, such that the hybrids expressed these genes at levels that are between the two parents but are statistically different from the mid-parent and parental levels. An assessment of AHP and BLP patterns applying more liberal criteria are presented below in section *Hybrid expression patterns outside of the parental range*.

In addition to using statistical tests to determine the types and frequencies of non-additive expression, we also utilized a variety of plots using d/a values to visualize the distribution of hybrid expression values relative to the parental expression levels. In our application of the d/a calculation (described in the Methods section), a d/a value of zero indicated additive hybrid expression, d/a values of 1 or -1 indicated hybrid expression levels equal to one of the parents, and d/a values > 1 or <-1 indicated hybrid expression levels outside of the parental range.

**Figure 6:**
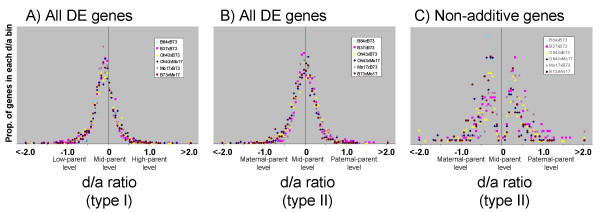
**Distribution of d/a values for Affymetrix differentially expressed genes**. Distributions of d/a ratios for differentially expressed genes based on Affymetrix microarray data. (A) d/a type I values indicate the hybrid expression levels relative to the low-parent and high-parent levels. The distributions are very similar for the six different hybrids. Hybrid expression patterns center approximately around the mid-parent level with very flat distributions outside of the parental range. (B) d/a type II values indicate the hybrid expression levels relative to the maternal-parent and paternal-parent levels. Again, all six hybrids exhibit similar distributions peaking around mid-parent levels, indicating no maternal or paternal expression biases. (C) The distributions of d/a type II values for the subset of differentially expressed genes that exhibited non-additive hybrid expression profiles. The distributions indicate that the non-additive patterns for most genes are still within the parental range, and are oftentimes observed near the mid-parent (additive) values.

We performed the d/a calculations in two different ways (see Methods for calculation details). The first d/a calculation (hereafter termed 'd/a type I') assesses the hybrid expression levels relative to the high parent and low parent for each gene. The second d/a calculation (hereafter termed 'd/a type II') assesses the hybrid expression levels relative to the maternal parent and paternal parent, allowing for the identification of maternal or paternal effects on gene expression in the hybrid. The distributions of the d/a values for the six different inbred-hybrid groups were strikingly similar (Figure 6A–B). The d/a type I distribution for all six hybrids is centered at approximately zero, and the distribution tails consistently flattened within the parental range (between -1.0 and 1.0) (Figure 6A). We did note that the center of the d/a type I distribution is skewed slightly towards the low parent. We suspected that the slight deviation of d/a type I values from the mid-parent levels may be caused by technical rather than biological factors. We found that genes with lower expression signals exhibited greater deviation from zero than genes with high expression signals [see Additional file 5]. The d/a type I distribution for genes with at least one genotype signal > 10000 units exhibited no deviation from zero for all six hybrids [see Additional file 5]. These findings suggest that technical factors, such as a slightly non-linear dynamic range among the lower microarray signal intensities, may be causing the slightly skewed distributions.

Similar to the d/a type I findings, the d/a type II distributions also displayed a remarkably consistent distribution among the six hybrids patterns, as they each peaked at approximately zero and the tails flattened within the parental range (Figure 6B). There is no evidence for skewing of the d/a type II distribution, indicating that hybrid expression did not consistently favor the maternal or paternal parent. A previous study had noted an intriguing transcriptional parental effect in which the hybrid tissues collected from the immature ears of 16 different hybrids generally exhibited paternal-like expression patterns for genes that were more highly expressed in the maternal versus the paternal parent [15]. Genes that were more highly expressed in the paternal parent tended to exhibit mid-parent expression patterns in the hybrids [15]. We attempted to replicate the Guo et al. [15] analysis using the 'd/a type II' calculation on our Affymetrix dataset [see Additional file 5]. No such unidirectional skewing was observed in our dataset; the two gene subsets were equally skewed towards the respective low parent levels, which is simply a reflection of the low-parent skewing observed in Figure 6A. It is possible that the explanation for the differences between these two studies is because of the different tissues used, immature ears [15] versus seedlings (this study).

The d/a type II distribution for the subset of non-additive genes exhibited a bi-modal distribution, with the trough located around the additive d/a value of zero (Figure 6C). The distribution indicated that most non-additively expressed genes exhibited hybrid expression values between the parental levels, with only a small proportion of genes found outside of the d/a parental range of -1.0 to 1.0 (Figure 6C). This distribution confirms the conclusions based on statistical tests described above.

**Figure 7:**
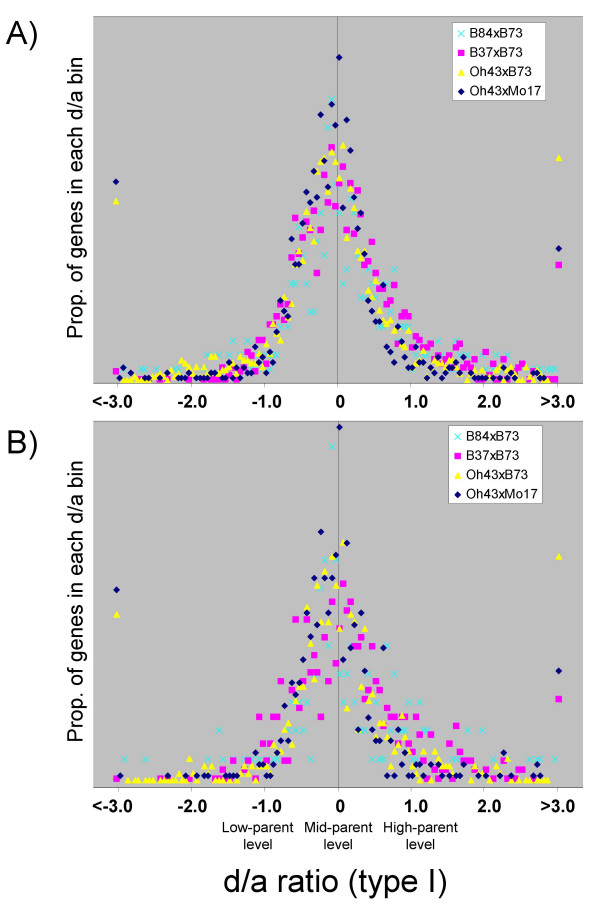
**Distribution of d/a values for 70-mer array differentially expressed genes**. Distributions of d/a (type I) ratios for differentially expressed genes based on the 70-mer oligonucleotide microarray data. (A) The d/a distributions for all differentially expressed genes. The distributions of the four hybrids are very similar to one another and peak at approximately zero, as was observed in Affymetrix microarray data. (B) The d/a distributions for the subset of differentially expressed genes that are also represented with features on the Affymetrix platform. The distributions are similar to those in (A). In both (A) and (B), the proportion of DE genes with d/a values above 3.0 or below -3.0 are all plotted as a single data point. The proportion of d/a values above 3.0 and below -3.0 for hybrid B84 × B73 plotted beyond the range of the displays and are not shown.

We also identified DE genes and calculated d/a type I values using the 70-mer oligonucleotide microarray data (see Methods for details on statistical analyses). The distribution of the d/a plots from 70-mer oligonucleotide microarray data are very similar to the plots generated from the Affymetrix data (Figure 7A). The d/a type I distribution for all four hybrids are similarly shaped, with each centered near zero (Figure 7A). However, the 70-mer oligonucleotide microarray d/a plots indicated that a substantial proportion of genes have hybrid expression levels outside of the parental range. This is evidenced by the fact that many of the genes exhibit d/a type I values greater than 1.0 or less than -1.0 (Figure 7A). In total, 20.6% of the DE patterns exhibited d/a values outside the parental range in the 70-mer oligonucleotide microarray data. By comparison, the Affymetrix d/a distributions were nearly flat outside of these values and only 1.3% of the DE patterns exhibited d/a values outside the parental range (Figure 6).

It is not clear why the two microarray platforms exhibited differences in the fraction of genes with d/a values outside the parental range. We considered the possibility that the different sets of genes represented on either platform may result in different rates of non-additive profiles. To address this, we generated a d/a plot (type I) of the 70-mer oligonucleotide microarray data using only the DE features that are also represented on the Affymetrix platform (Figure 7B). The resulting d/a distribution is very similar to the d/a distribution generated by all DE genes (Figure 7A), indicating that platform feature biases are not causing the differences in non-additive profiles observed between the microarray platforms.

It is important to remember that these d/a values are a composite of multiple biological replicates and they do not include estimates of variation. A closer inspection of several genes with d/a values above 1.0 or below -1.0 revealed that while the average d/a values are outside the parental range, they are often not statistically significant. We estimated the degree of variation within each platform by comparing the signal intensity variation among the biological replicates within each genotype. For each DE gene, we divided the standard deviation of the three biological replicates by the mean of the three biological replicates. These calculations indicated that the 70-mer oligonucleotide microarray data generated approximately twice as much signal variation among replicates than the Affymetrix platform [see Additional file 6]. This higher level of signal variation likely contributes to the wider distributions of d/a values observed in Figure 7.

Overall, the Affymetrix data d/a plots indicated that the hybrid expression distributions were similar for all six hybrids, with peaks at approximately zero and very few genes exhibiting hybrid expression patterns outside of the parental range (d/a > 1.0 or <-1.0) (Figure 6). This is in strong agreement with the clustered heat maps [see Additional file 4] and statistical analyses of additivity (Table 1). In general, the hybrids exhibited additive expression and the majority of genes with non-additive expression still exhibited expression levels within the parental range.

### Hybrid expression patterns outside of the parental range

The analyses of Affymetrix microarray data described in the previous section applied relatively stringent statistical significance parameters. The Affymetrix results identified 5020 DE patterns among the parents and hybrids of six crosses, however only 15 hybrid gene expression patterns were found to be significantly outside of the parental ranges. Several other groups have reported observing much higher frequencies of hybrid expression outside the parental range [21-23]. In this section, we have applied more liberal statistical significance and fold-change thresholds to the Affymetrix data in order to identify hybrid expression patterns outside of the parental range that may have been missed when applying the stringent statistical criteria.

The number of DE genes was substantially increased when applying an ANOVA FDR < 0.15 (as opposed to 0.05 in Table 1). This identified a total of 13,280 DE patterns among the six hybrids (Table 2 and 3). We then determined the number of patterns that exhibited expression above (318) or below (538) the parental range. Only 6.4% of all DE patterns exhibited expression levels outside the parental range. Of the 856 examples of expression outside the parental range, only 221 patterns are statistically different from the near-parent levels (Table 2 and 3). These 221 patterns represent 213 genes (eight genes exhibited AHP or BLP in two of the six inbred-hybrid groups). The majority of these genes showed less than 1.1-fold differences from the near-parent, and successively higher fold-change stringency thresholds rapidly filtered the remaining genes; only nine genes showed greater than 2-fold changes outside of the parental range (Table 2 and 3). These data indicate that for the vast majority of AHP and BLP genes, the expression divergence from near-parent levels is relatively small. Several methods were used to validate these AHP and BLP expression patterns. We began by comparing the d/a values for these 221 examples of AHP or BLP expression in the Affymetrix data to the d/a values for these genes in the 70-mer oligonucleotide microarray data [see Additional file 7]. The 70-mer oligonucleotide microarray data supported AHP or BLP expression for 53 of the 127 genes (42%) with available data. Thus, the 70-mer oligonucleotide microarray data validated some of the examples of AHP or BLP expression, but also indicated that some of these profiles may be false-positives. Quantitative real-time PCR analyses validated the AHP or BLP expression for six of eight of the genes tested (these 8 genes were selected due to the availability of good sequence and the ability to design gene-specific primers) [see Additional file 7]. We also noted that many of the 213 AHP or BLP genes tend to exhibit low levels of AHP or BLP expression in multiple hybrids, suggesting potential conservation of the AHP or BLP patterns [see Additional files 7 and 8]. Our results indicate that a small fraction of genes display significant AHP and BLP expression in hybrid maize seedlings. Furthermore, comparisons of the different inbred-hybrid combinations provide evidence that many of these genes are consistently expressed outside of the parental range across different hybrid lines.

**Table 2:** Identification of above-high parent (AHP) expression patterns

		B84 × B73	B37 × B73	Oh43 × B73	Oh43 × Mo17	Mo17 × B73	B73 × Mo17	Total (%)
	#DE Genes*	920	1979	2851	2430	2570	2530	13280
	#DE Genes F1>HP**	9	32	139	62	24	52	318 (2.39%)
	#DE Genes F1>HP (p < 0.05)***	1	8	47	14	7	16	93 (0.70%)

AHP fold-change	#Sig. genes w/ F1/HP 1.0–1.1	1	3	16	8	5	6	39 (0.29%)
	#Sig. genes w/ F1/HP 1.1–1.2	0	3	24	2	1	6	36 (0.27%)
	#Sig. genes w/ F1/HP 1.2–1.5	0	0	5	2	0	2	9 (0.07%)
	#Sig. genes w/ F1/HP 1.5–2.0	0	1	0	1	1	1	4 (0.03%)
	#Sig. genes w/ F1/HP > 2.0	0	1	2	1	0	1	5 (0.04%)

*Based on FDR < 0.15 and at least one genotype (parent and/or hybrid) average microarray signal > 50

**Subset of DE genes with microarray signal intensities outside of the parental range (AHP or BLP, respectively)

***Subset of DE genes with microarray signal intensities significantly outside of the parental range (based on one-tailed t-tests)

**Table 3:** Identification of below-low parent (BLP) expression patterns

		B84 × B73	B37 × B73	Oh43 × B73	Oh43 × Mo17	Mo17 × B73	B73 × Mo17	Total (%)
	#DE Genes*	920	1979	2851	2430	2570	2530	13280
	#DE Genes F1<LP**	9	98	150	111	86	84	538 (4.05%)
	#DE Genes F1<LP (p < 0.05)***	1	26	41	31	20	9	128 (0.96%)

BLP fold-change	#Sig. genes w/ LP/F1 1.0–1.1	0	6	9	12	6	5	38 (0.29%)
	#Sig. genes w/ LP/F1 1.1–1.2	1	10	24	11	7	2	55 (0.41%)
	#Sig. genes w/ LP/F1 1.2–1.5	0	8	5	6	7	1	27 (0.20%)
	#Sig. genes w/ LP/F1 1.5–2.0	0	1	1	2	0	0	4 (0.03%)
	#Sig. genes w/ LP/F1 > 2.0	0	1	2	0	0	1	4 (0.03%)

*Based on FDR < 0.15 and at least one genotype (parent and/or hybrid) average microarray signal > 50

**Subset of DE genes with microarray signal intensities outside of the parental range (AHP or BLP, respectively)

***Subset of DE genes with microarray signal intensities significantly outside of the parental range (based on one-tailed t-tests)

### Gene ontology analyses

**Figure 8:**
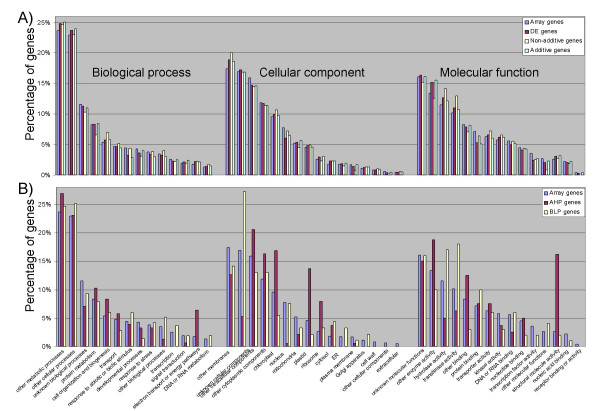
**Distribution of DE, AHP and BLP genes among gene ontologies**. (A) The distributions of GO terms assigned for the entire Affymetrix microarray, the differentially expressed genes, and the subsets of additive and non-additive differentially expressed genes are compared. (B) The distributions of GO terms assigned for the entire Affymetrix microarray, the AHP subset and the BLP subset are compared. In both (A) and (B), the GO terms are ordered on the graph from highest frequency on the microarray (left) to lowest frequency on the microarray (right) within the Biological process, Cellular component, and Molecular function categories, respectively.

We compared the relative representation of gene ontology (GO) categories for the DE genes versus the total number of probe sets present on the Affymetrix microarray. For this analysis, the DE genes from the stringent Affymetrix analysis (FDR < 0.05, and minimum signal and fold-change filters; see Methods) were combined from the six inbred-hybrid groups. We did not identify any substantial divergence or overrepresentation of any specific GO annotation in the DE genes (Figure 8A). We also tested the full set of genes with additive or non-additive expression and did not find enrichment for any GO annotations within these lists of genes (Figure 8A). The relative proportion of each category approximately matched the proportion present on the microarray, suggesting that differential expression and additivity occur at equal rates for all functional types of genes.

We also compared the GO representation for the AHP and BLP genes versus the total number of probe sets present on the Affymetrix microarray. In this case, we did identify some obvious over- and under-represented categories (Figure 8B). Most obviously, the AHP genes appeared to be over-represented by electron transport and energy pathway processes, plastid and ribosome components, and structural molecular functions. The AHP genes were under-represented in nucleus components and several molecular function categories, including transcription factor activities. Generally, the BLP genes exhibited less frequent over- or under-representations than the AHP genes. Over-represented BLP categories included hydrolase and transferase molecular functions.

The biological meaning of the over- and under-represented AHP and BLP categories remains unclear. The function of these gene classes may be particularly important in conferring heterosis. However, because the number of AHP and BLP genes is relatively small (only ~1.6% of the total microarray features), frequency analyses of these genes are more susceptible to sampling and stochastic effects.

## Discussion

### Linking maize genetic diversity and transcriptional variation

It is especially important to recognize that genetic and transcriptional assessments of natural variation apply different experimental procedures and analysis tools. Sequence-based genetic diversity studies utilize a stable character for scoring variation, typically DNA sequence polymorphisms. Studying transcriptional diversity involves measuring an unstable unit, mRNA, that is subject to change based on developmental and environmental cues. Multiple sources of variation in gene expression datasets may increase the measurement variance among replicates, thereby reducing statistical power.

In the present study, we compared the transcriptional diversity of six different maize inbred-hybrid combinations. We found that the number of DE genes identified for each inbred-hybrid group strongly correlated with the genetic diversity between inbred lines, as estimated by SNP-based sequence analyses [23]. A comparison of the number of DE genes for each of the 10 possible pairs of inbred genotypes also revealed a strong correlation between transcriptional and genetic diversity (data not shown). Our previous work using allele-specific expression assays indicated that maize intraspecific transcriptional variation is primarily driven by *cis*-acting sources of variation [17,26]. It is possible that increased levels of sequence polymorphism linked to genes may be at least partially responsible for the higher rates of transcriptional variation observed in more genetically distant inbreds. Indeed, the intergenic space in the maize genome is known to be highly polymorphic among inbred lines [12,28], and these structural and nucleotide polymorphisms may drive transcriptional variation of certain maize genes.

### Implications of non-additive expression patterns

We were also interested in identifying possible links between transcriptional profiles and heterotic performance. A higher number of differentially expressed genes were identified in the inbred-hybrid combinations representing more distantly related genotypes. The hybrids derived from more genetically diverse inbred parents exhibited higher numbers of both additive and non-additive gene expression patterns. However, the proportion of non-additive hybrid expression profiles among the DE genes was similar for all six hybrids. Additionally, the relative proportions of genes that display different types of non-additive expression were similar in the six hybrids. These data suggest that the prevalence of non-additive expression in seedling tissue is not correlated with different heterosis levels.

It is tempting to infer that non-additive hybrid expression patterns imply novel hybrid regulation or may be associated with heterosis. However, it is important to consider that non-additive expression patterns include both predictable and unpredictable patterns. Using the "expression level" as a phenotype, we can describe non-additive expression patterns using dominance terminology. The genes with between-parent non-additive expression can be described as partially dominant while genes with HP or LP expression can be described as dominant. Classical genetics provides many examples of partial or complete dominance in an F_1 _hybrid and in many cases the molecular mechanisms for this type of inheritance have been determined. In our study, even when we applied liberal statistical criteria for DE gene identification, > 98% of the non-additively expressed genes exhibited expression phenotypes that could be described as partial or complete dominance.

Many studies of intraspecific F_1 _hybrid gene expression have focused upon the identification of genes with expression levels outside the parental range, including studies in Drosophila [29], Arabidopsis [14] and oyster [30], among others. Such patterns (termed AHP or BLP in this study) have often been described as over- or under-dominant. These are unpredictable hybrid expression patterns and may be caused by novel hybrid-specific regulatory mechanisms. The importance of AHP or BLP expression patterns in heterosis is unclear. It is possible that the AHP or BLP expression patterns may play a role in driving heterosis. However, it is also possible that the AHP or BLP expression patterns are a consequence, not a cause, of heterosis.

The previous study by Swanson-Wagner et al. [11] had documented evidence that while additive expression was most common, all potential modes of hybrid gene expression were observed in the B73 × Mo17 hybrid. In this study we report similar findings and extend this analysis to additional hybrids that exhibit different levels of genetic diversity. The number of AHP or BLP genes reported in maize hybrid expression profiles has varied widely from essentially none [17] to a small proportion of genes [11,15] to a larger proportion of genes [18,23] and even up to > 50% of DE genes [21]. There are several potential explanations for this discrepancy. It could be that different tissues or developmental stages exhibit different levels of AHP and BLP expression. Alternatively, it could be that different expression profiling technologies, sampling methodologies or experimental designs influence the discovery of AHP and BLP expression patterns, as described by Cui et al. [31] and Rottscheidt and Harr [32]. Indeed, using the same RNA samples across platforms, we found substantially more AHP and BLP patterns on the 70-mer oligonucleotide microarray platform than on the Affymetrix platform. However, real-time PCR rarely validated the 70-mer oligonucleotide microarray AHP and BLP patterns (2/12 genes; data not shown), but more frequently validated the Affymetrix AHP and BLP patterns (6/8 genes). If we assume that AHP and BLP expression patterns are quite rare, then greater degrees of technical variation in an expression profiling platform may lead to higher numbers of false-positive AHP or BLP observations.

### Complications in predicting heterosis

In this study, we investigated the heterotic responses of 25 different maize F_1 _hybrids across five different traits, plant height, days to flowering, seed weight, seedling height and seedling biomass. Our goal was to ascertain whether heterosis for any particular trait was predictive of heterosis for another trait. Furthermore, we wanted to observe the relationship between heterosis and parental genetic distance for each trait.

A major goal of this study was to compare the types of expression variation observed in hybrids with differing levels of heterosis. In order to perform this experiment we wanted to ascertain whether certain hybrids would show higher or lower levels of heterosis for a variety of traits. However, we found that there is generally a lack of correlation for heterosis levels among different traits. Few hybrids appeared to consistently exhibit either high or low relative heterosis among traits. This suggests that heterosis is not an organism wide phenomenon but instead is trait-specific, and is likely controlled by partially non-redundant sets of genes for different traits.

Previous studies have found that genetic distance between inbred parents is correlated with grain yield heterosis in maize when the parental lines are closely related but that this correlation breaks down when the parental lines are distantly related [5]. Our analysis of non-yield traits in a relatively small number of hybrid genotypes concurs with the previous studies on the correlation between yield heterosis and genetic distant. We found that genetic distance was an inconsistent predictor of heterosis. Only one of five traits (seedling biomass) was found to have a significant correlation between genetic distance and heterosis. In general, hybrids derived from closely related parents had relatively low levels of heterosis. However, hybrids derived from distantly related parents displayed a range of heterotic responses, including high and low BPH values. As in studies of yield heterosis, this means that genetic distance can often be used to predict poorly performing hybrids but has weak power to predict superior hybrids. Hamblin et al. [24] suggested that the difficulty in predicting hybrid performance for more distantly related parents may be due in part to difficulties in accurately assessing genetic distance for more distantly related inbreds.

## Conclusion

This study indicates that there is a strong correlation between genetic diversity and transcriptional variation among maize inbreds. However, the degree of genetic or transcriptional variation between the inbred parents appears to be an inconsistent predictor of hybrid heterosis, depending on the trait of interest. The frequency and patterns of non-additive hybrid expression profiles appear to be similar among different hybrids. Together, these data suggest that maize hybrid heterosis may be more influenced by the additive complementation of transcriptional variation than by novel non-additive expression states.

## Methods

### Plant growth and phenotyping

Fifteen inbred lines and twenty-five hybrids were grown on the St. Paul campus Agricultural Experiment Station during the Summer of 2006. One row of each genotype was planted in each of two randomized complete blocks; the block planting dates were separated by 12 days. All plants that reached full maturity were scored for three traits; plant height, flowering time and weight of 50 seeds. The plants were monitored daily following tassel development and the flowering date for each plant was recorded as the first day the tassels shed pollen. Thereafter, mature plant heights were measured as the distance from the ground to the top of the tassel. Open-pollinated ears were harvested from each plant and dried; 50 seeds were collected and weighed from each ear to measure average seed mass. The mean values for each trait were calculated for each genotype within each biological replicate; 70% trimmed means were used for the height and days to flowering traits to control for outliers. The trait means and standard deviations were calculated from the two biological replicate means and were subsequently used to calculate the percentage heterosis for each trait. The percentage better parent heterosis (BPH) was calculated for each biological replicate as:

%BPH = [(Hybrid mean – Better-parent mean)/Better-parent mean] × 100

The overall %BPH mean and standard deviation was calculated based on the two biological replicate %BPH values. Heterosis for days to flowering is considered here as a measure of earliness, and thus BPH represents the percentage of time hybrid plants flowered before the earlier-flowering parent.

The same genotypes were planted in 7 1/2" azalea pots and grown in standard greenhouse conditions for 11 days. For each biological replicate, a total of eighteen seedlings were grown (six seedlings each in three pots). The pots were placed in a randomized design within the greenhouse. Three biological replicates of this experiment were planted in succession. After 11 days of growth the seedlings were scored for above-ground plant height by measuring from the base of the plant to the tip of the longest extended leaf. Additionally, above-ground seedling tissues were harvested, dried, and then weighed for biomass measurements. The mean values for height and biomass were calculated for each genotype within each biological replicate. Percentage BPH was calculated using the mean values across biological replicates, as described above.

BPH measurements were plotted against the intra- or inter-heterotic group status of each cross and the genetic distance between parents of each cross. The intra- or inter-heterotic group status of each cross was based on the classifications by Flint-Garcia et al. [33]. The Nei genetic distance values are based on data from 847 SNP markers [24].

### Affymetrix microarray analyses

RNA was isolated from above ground tissue of 11-day old maize seedling for five maize inbreds (B37, B73, B84, Mo17 and Oh43) and four hybrids (B37 × B73, B84 × B73, Oh43 × B73 and Oh43 × Mo17). Three biological replicates were grown using standard greenhouse conditions (1:1 mix of autoclaved field soil and MetroMix; 16 hours light and 8 hours dark; daytime temperature of 30°C and night temperature of 22°C) and sampled on the 11^th ^day after planting between 8:00 and 9:00 am. The plants were cut immediately above the highest brace root, thus all above-ground tissues and meristems were collected. Each biological replicate consisted of pooled tissue from eight different seedlings of the same genotype. Total RNA samples were isolated using TRIzol (Invitrogen, Carlsbad, CA) and purified using the RNeasy system (QIAGEN, Valencia, CA). RNA quantity and quality were assessed using the Nanodrop spectrophotometer (Nanodrop Technologies, Montchanin, DE) and agarose gel electrophoresis.

Affymetrix microarray hybridizations using the Maize GeneChip were performed for RNA samples from three biological replicate samples for each genotype. RNA collection, labelling and hybridization followed published methodologies [17]. The Affymetrix microarray data was deposited in the Gene Expression Omnibus (GEO) under accession number GSE10236. Affymetrix microarray data previously generated with the same experimental design on genotypes B73, Mo17, B73 × Mo17 and Mo17 × B73 [17] (GEO accession number GSE8174) were included in the analyses for purposes of further comparison.

Microarray statistical analyses were performed with each parent-hybrid group. For example, the genotypes B37, B73 and B37 × B73 were normalized and analyzed together, the genotypes Oh43, B73 and Oh43 × B73 were normalized and analyzed together, and so on for the six different parent-hybrid groups. Data normalization between microarrays was performed using GC-RMA, and a per-gene normalization was applied to the resulting values using GeneSpring version 7.2 software. Genes that were differentially expressed among genotypes were identified by performing a one-way ANOVA on the normalized data using a parametric test with no assumption of equal variance. A Benjamini and Hochberg correction for multiple testing was applied using a false-discovery rate (FDR) of 0.05. We removed genes that did not exhibit at least one genotype with an average microarray signal greater than 50 units and genes that did not exhibit at least a 1.2-fold change between any two of the three genotypes. These filters were imposed to remove genes with very minor differential expression or genes with little evidence for expression. Thus, genes that exhibited a FDR < 0.05 and passed the minimum signal and fold change thresholds were determined to be differentially expressed.

Subsequent analyses of the differentially expressed genes focused on assessing the expression levels of hybrid versus parental genotypes. Hierarchical clustering of gene expression profiles were conducted using GeneSpring software. A statistical test for non-additive hybrid expression levels was performed by comparing the inbred midparent expression levels versus the hybrid expression levels. A two-tail homoscedastic *t*-test was performed and all genes with *P *< 0.05 were considered to be non-additively expressed.

The d/a ratios were calculated for all differentially expressed genes using two different methods, which we have termed type I and type II for purposes of clarification. Type I d/a values were calculated as described in Stupar et al. [20] and were used to compare the hybrid expression relative to the high parent and low parent for each gene. Briefly, to calculate the type I d/a, the d value is calculated as the hybrid signal minus the average signal of the two parents, and the a value is calculated as the high parent signal minus the average signal of the two parents. Genes with d/a values equal to 0.0 exhibit additive expression compared to the parents. Genes with d/a values equal to 1.0 or -1.0 exhibit hybrid expression levels equal to the high parent (HP) or low parent (LP), respectively. Genes with d/a values greater than 1.0 or less than -1.0 indicated genes with hybrid expression levels above the high-parent (AHP) or below the low-parent (BLP), respectively. Statistical confirmation of AHP or BLP patterns was determined by a one-tailed homoscedastic *t*-test of the hybrid expression values versus the high-parent or low-parent values, respectively; genes with *P *< 0.05 were considered valid AHP or BLP calls. Type II d/a values were used to compare the hybrid expression relative to the maternal and paternal parent for each gene. To calculate the type II d/a, the d value is calculated as the hybrid signal minus the average signal of the two parents, and the a value is calculated as the paternal parent signal minus the average signal of the two parents. Genes with d/a values equal to 0.0 exhibit additive expression compared to the parents. Genes with d/a values equal to 1.0 or -1.0 exhibit hybrid expression levels equal to the paternal parent or maternal parent, respectively. Genes with d/a values greater than 1.0 or less than -1.0 indicated genes with hybrid expression levels outside of the parental range.

A second set of analyses were conducted on the Affymetrix data to liberalize our search for genes with hybrid AHP or BLP expression patterns. The ANOVA were performed as described above, however a FDR threshold of 0.15 was applied to identify differentially expressed genes. Homoscedastic one-tail *t*-tests (*P *< 0.05) of the hybrid expression levels versus the high parent or low parent were used to identify hybrid genes with AHP or BLP patterns, respectively. The magnitude of the AHP and BLP expression patterns were estimated by calculating the fold change of the hybrid versus the high parent or low parent, respectively.

Gene ontology analyses of the DE, AHP and BLP gene subsets were performed as described in Makarevitch et al. [34].

### 70-mer oligonucleotide microarray analyses

The same RNA samples from the five maize inbreds (B37, B73, B84, Mo17 and Oh43) and four hybrids (B37 × B73, B84 × B73, Oh43 × B73 and Oh43 × Mo17) were also hybridized to the maize 70-mer oligonucleotide microarray developed at the University of Arizona [27]. This array contains 43,537 unique 70-mer oligonucleotide features. Two-color microarray hybridizations were performed according to a reference design over the three biological replicates from each genotype. The 70-mer oligonucleotide microarray data was deposited in GEO under accession number GSE10542.

Hybridization target was prepared using a protocol [35] similar to that of Eberwine [36] which utilizes an oligo dT primer that incorporates a T7 viral promoter to linearly increase mRNA concentration by in vitro transcription. The target amplification protocol utilized the Ambion Aminoallyl Message Amp II kit (Catalog # 1751, Ambion, Austin TX), which had been optimized to utilize reduced reaction volumes. Sample RNAs were labelled with Cy3 dye and the pooled reference samples were labelled with Cy5 dye. Prior to hybridization, the slides were prepared as described previously by Gardiner et al. [27]. The 70-mer arrays were hybridized on a Tecan 4800 HS Pro hybridization station (Tecan Services Inc., Durham, NC) which is capable of processing 12 arrays in a single hybridization run. The arrays were scanned on an Axon 4100 AL scanner (Molecular Devices Corporation, Sunnyvale, CA) immediately after being hybridized. Raw expression data were generated from the resulting TIF images using the GenePix 6.0 software package (Molecular Devices Corporation, Sunnyvale, CA)

Initial microarray statistical analyses were performed in order to compare the Affymetrix and 70-mer oligonucleotide microarray results. The (median – median background) Cy3 70-mer microarray signal intensities were normalized with each parent-hybrid group using the per chip normalize to 50^th ^percentile and the per gene normalize to median parameters in Genespring version 7.2 software. We identified the 70-mer microarray features that matched the differentially expressed probe sets in the Affymetrix dataset. For these genes, the log_2 _fold-change expression ratios between inbred genotypes were calculated for both microarray platforms. These ratios were compared across the Affymetrix and 70-mer microarray datasets to determine the correlation among platforms.

A second set of statistical analyses were performed on the 70-mer oligonucleotide microarray data to identify genes that were differentially expressed among genotypes within each parent-hybrid group. For these analyses, spot values from the raw data were flagged if any of the following were observed: > 30% of the pixels were saturated in either channel, spot diameter < 70 um in either channel, or spot was not found by the scanning software. Genes in which flags were found in any of the biological replicates of at least two genotypes were removed from further analyses. Additionally, genes in which the average raw signal intensity was < 200 units in the most intense genotype were also removed. Following these filtration steps, ~40% of the spot features remained for each parent-hybrid group. Data from these remaining features were normalized using the per chip normalize to 50^th ^percentile and the per gene normalize to median parameters in Genespring version 7.2 software, as described above. Genes that were differentially expressed among genotypes were identified by performing a one-way ANOVA on the normalized data using a parametric test with no assumption of equal variance. A Benjamini and Hochberg correction for multiple testing was applied using a false-discovery rate (FDR) of 0.10. Genes that did not exhibit at least a 2-fold change between any two of the three genotypes were removed from further analyses. Additionally, features that exhibited statistically significant changes in the Cy5 reference channel (FDR < 0.10) or showed a high degree of variation in the Cy5 reference channel ((signal standard deviation)/(signal mean) > 0.50) were also removed. Using this series of statistical tests and quality control measures, the following numbers of features were found to be differentially expressed among genotypes on the 70-mer oligonucleotide microarray: parent-hybrid group B37, B73, B37 × B73: 1,555; B84, B73, B84 × B73: 430; Oh43, B73, Oh43 × B73: 1,847; Oh43, Mo17, Oh43 × Mo17: 1,183. The d/a ratios were calculated for these genes as described above in the *"Affymetrix microarray analyses" *section.

### Expression validation using Real-Time qPCR

The same RNA samples used for microarray analyses were also used for real-time qPCR in an attempt to validate some of the AHP and BLP expression patterns. A set of eight genes with AHP or BLP expression levels at least 1.5 fold outside the range of the parents were selected from the Affymetrix analyses (specific genes are indicated in Additional file 7). Another 12 genes with AHP or BLP expression levels at least 1.5 fold outside the range of the parents in the 70-mer oligonucleotide microarray analysis of Oh43 × B73 hybrids were also selected. These genes were selected based on our ability to design effective, gene-specific primers for real-time qPCR analyses. 2.5 μg of total RNA was treated with RQ1 DNase (Promega, Madison, WI) according to manufacturer's instructions to remove contaminating DNA. RNAs were immediately cooled on ice following DNAse digestion, mixed with 1 μg oligo dT (Promega) and heated to 70°C for 10 minutes, followed by 1 minute on ice. First strand cDNA synthesis was performed using 1 μl M-MLV reverse transcriptase according to the manufacturer's instructions (Promega). This reaction was incubated at 42°C for 50 minutes followed by 70°C for 15 minutes. 1 μl of cDNA was used as template in real-time qPCR reactions, containing 10 μl 2× SYBR Green PCR Master Mix (Applied Biosystems, Foster City, CA), 1 μl each of forward and reverse primer and 7 μl water. Reactions were performed using the 7900HT Real-Time PCR System (Applied Biosystems) with the following cycling parameters: 95° for 10', 40 cycles of 95° for 30", 60° for 30", 72° for 30", followed by a disassociation stage (melting curve analysis). Threshold value was empirically determined based on the observed linear amplification phase of all primer sets. Sample cycle threshold (Ct) values were standardized for each template based on a GAPC control primer reaction, and the comparative Ct method [37] was used to determine the relative transcript abundance of each gene.

## Authors' contributions

RMS conceived of the study, participated in its design, collected heterosis phenotype data, carried out the Affymetrix microarray hybridizations, performed all microarray data analyses and drafted the manuscript. AGO performed the heterosis phenotype data collection and analysis. WJH carried out the real-time PCR analyses. VLC and JMG were responsible for the 70-mer microarray hybridizations and scanning and helped draft the manuscript. NMS conceived of the study, participated in its design, and helped draft the manuscript. All authors read and approved the final manuscript.

## Supplementary Material

Additional file 1 Phenotypic measurements for five traits in 15 inbreds and 25 hybrids. Click here for file

Additional file 2 Genetic distance between inbreds and BPH values for hybrids. Nei SNP genetic distance values are shown for 21 inbred pairwise comparisons. BPH for five traits are also shown for the 21 corresponding hybrids, and four other hybrid combinations. Click here for file

Additional file 3 Pearson's R values for genotype BPH among traits. Correlations of BPH across the five traits are given. The primary data used to compute these correlations are given in Additional files 1 and 2. Click here for file

Additional file 4 Clustering analysis of differentially expressed genes. Clustering analysis to compare inbred-hybrid expression patterns. Click here for file

Additional file 5 Distribution of d/a ratios for specific sub-groups. Comparison of d/a distributions for specific subsets of genes. Click here for file

Additional file 6 Estimate of microarray signal variation between biological replicates of each genotype on the Affymetrix and 70-mer oligonucleotide microarray platforms. Click here for file

Additional file 7 d/a values across genotypes for genes identified as AHP or BLP in at least one hybrid using liberal criteria. The d/a values for each of the six hybrid genotypes are shown for genes exhibiting AHP or BLP in at least one hybrid. This display is used to identify genes with consistent patterns of AHP or BLP expression across hybrids, based on Affymetrix data. Cross-validation data from the spotted microarray and real-time PCR platforms are also shown. Click here for file

Additional file 8 Clustering analysis of genes with AHP and BLP profiles. Comparison of AHP and BLP profiles across multiple hybrid genotypes. Click here for file
