# Supplementary material for: Gene expression analyses in maize inbreds and hybrids with varying levels of heterosis
Source: BMC Plant Biol. 2008 Apr 10;8:33. doi: 10.1186/1471-2229-8-33 (PMC2365949; doi:10.1186/1471-2229-8-33)
Supplement: Additional file 4 — Clustering analysis of differentially expressed genes. Clustering analysis to compare inbred-hybrid expression patterns. [file 1471-2229-8-33-S4.pdf]

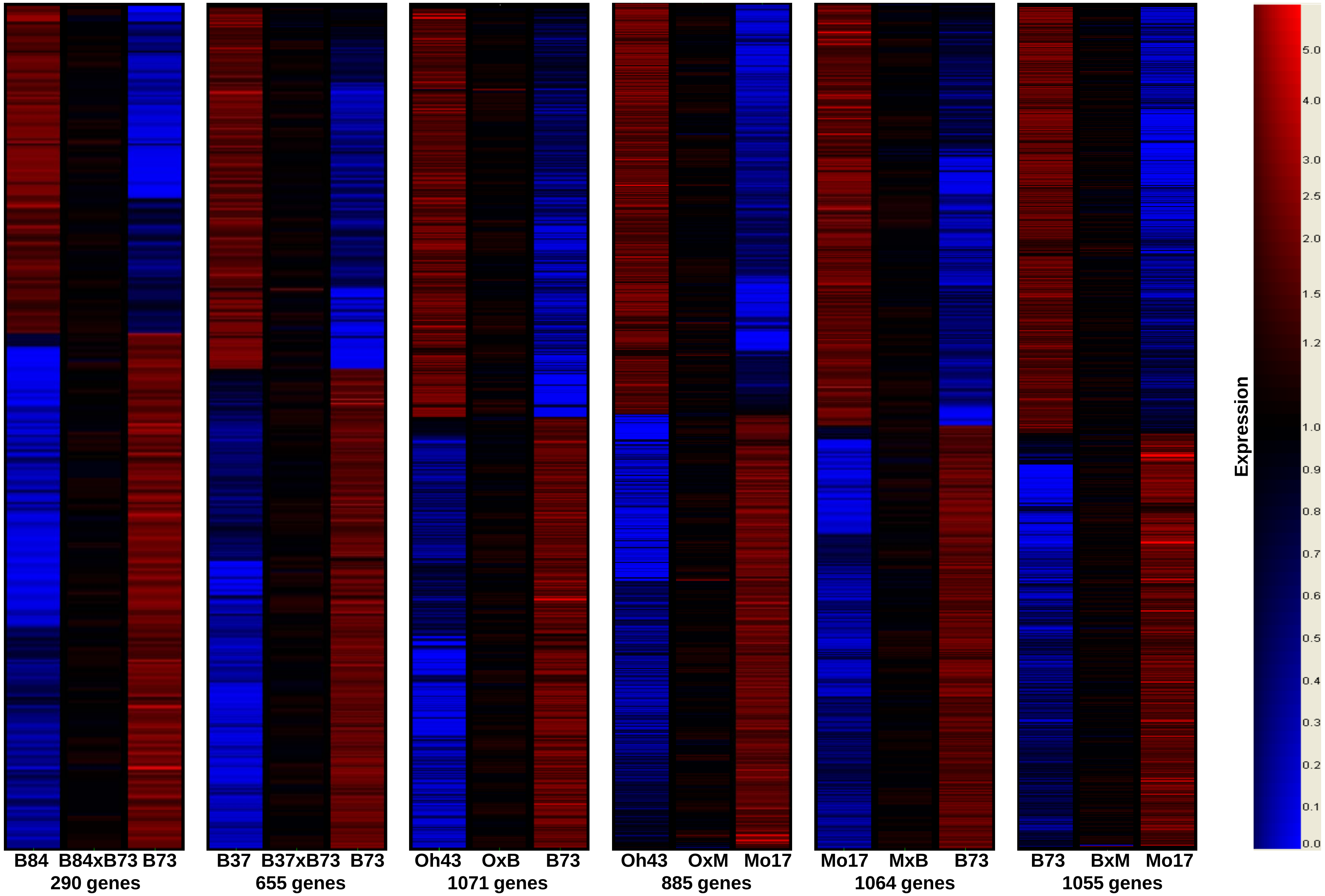

Additional file 4. Clustering analysis of differentially expressed genes. Heat map clusters depicting the gene expression levels of inbred relative to hybrids in the Affymetrix dataset. Genes determined to be differentially expressed (FDR < 0.05, and additional quality control filters; see Materials and Methods) among the inbred-hybrid combinations are shown. The numbers of differentially expressed genes for each group are shown at the bottom of the figure. Red indicates high relative expression and blue indicates low relative expression, while black indicates the average relative expression for each gene profile. The clusters indicate that all six hybrid predominantly exhibit mid-parent (additive) levels of expression.
