# Supplementary material for: Gene expression analyses in maize inbreds and hybrids with varying levels of heterosis
Source: BMC Plant Biol. 2008 Apr 10;8:33. doi: 10.1186/1471-2229-8-33 (PMC2365949; doi:10.1186/1471-2229-8-33)
Supplement: Additional file 5 — Distribution of d/a ratios for specific sub-groups. Comparison of d/a distributions for specific subsets of genes. [file 1471-2229-8-33-S5.pdf]

A)

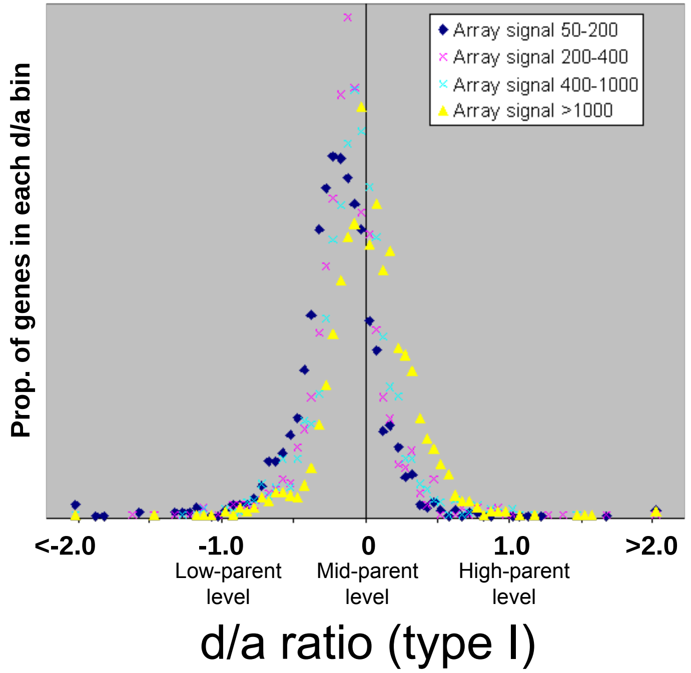

B)

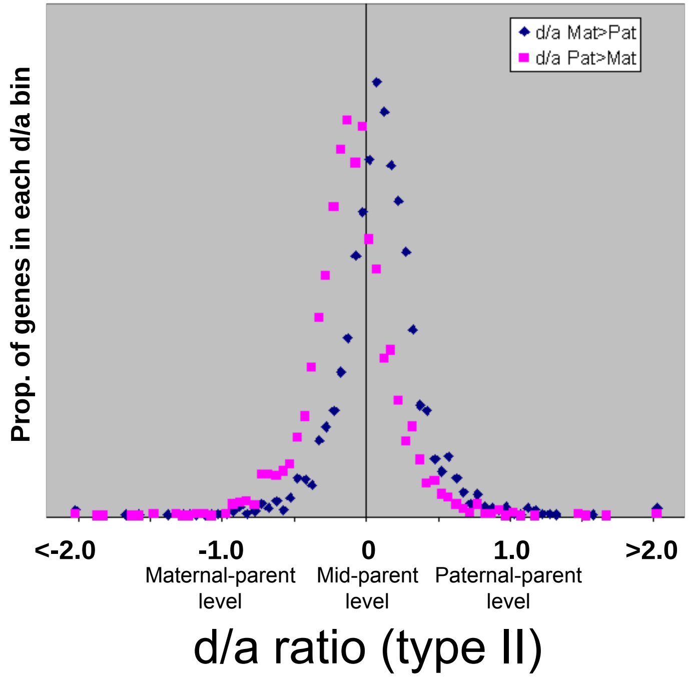

Additional file 5. Distribution of d/a ratios for specific sub-groups. Distributions of d/a ratios for differentially expressed genes based on Affymetrix microarray data. (A) The d/a type I distributions have been sub-grouped by expression level within each inbred-hybrid combination. The measurement range of each sub-group is determined by the genotype with the highest microarray signal in each inbred-hybrid combination. d/a values of 0.0 indicate mid-parent hybrid expression levels, whereas values of 1.0 or −1.0 indicate high-parent or low-parent hybrid expression levels, respectively. It is evident that genes exhibiting low expression tend to exhibit stronger hybrid skewing towards the low-parent, suggesting that the skewing observed in Figure 4A (main text) may be caused by a non-linear dynamic range in the low levels of the signal detection range. (B) d/a type II values for genes that are more highly expressed in the maternal parent and genes that are more highly expressed in the paternal parent. The data from all six hybrids are combined in these distributions. The slight skewing towards either parental level reflects the skewing towards the low-parent observed in Figure 4A, believed to be caused by technical sources of variation (see part (A); see text).
