# Supplementary material for: Gene expression analyses in maize inbreds and hybrids with varying levels of heterosis
Source: BMC Plant Biol. 2008 Apr 10;8:33. doi: 10.1186/1471-2229-8-33 (PMC2365949; doi:10.1186/1471-2229-8-33)
Supplement: Additional file 8 — Clustering analysis of genes with AHP and BLP profiles. Comparison of AHP and BLP profiles across multiple hybrid genotypes. [file 1471-2229-8-33-S8.pdf]

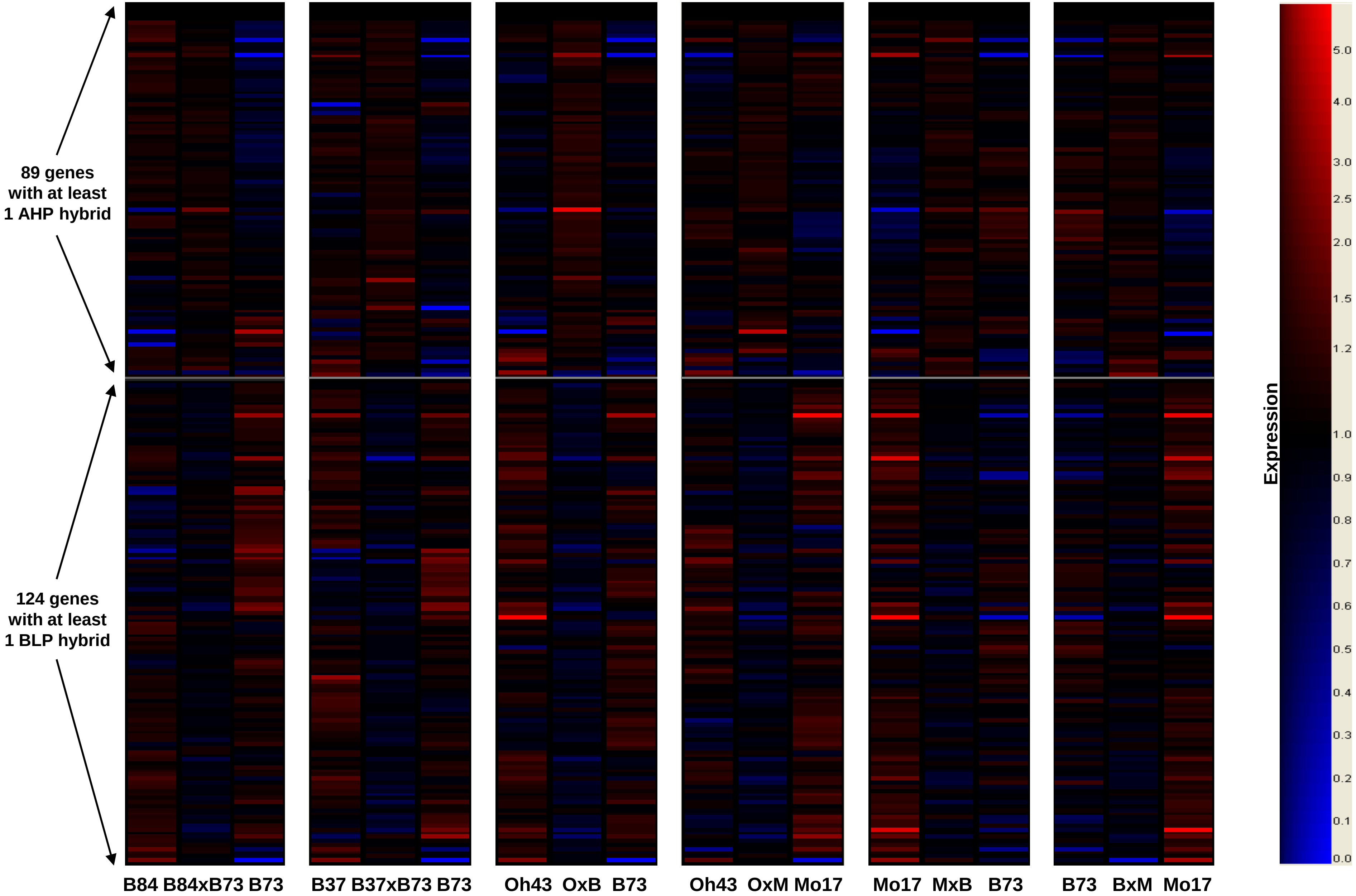

Additional file 8. Clustering analysis of genes with AHP and BLP profiles. Heat map clusters depicting the expression levels of genes in the Affymetrix dataset showing evidence for AHP or BLP expression in at least one inbred-hybrid group. The 213 genes determined to be AHP or BLP in at least one inbred-hybrid combination using liberal statistical thresholds (FDR < 0.15, and d/a value either greater than 1.0 or less than −1.0) are shown. Red indicates high relative expression and blue indicates low relative expression, while black indicates the average relative expression for each gene profile. The d/a values and statistical significance tests among the six inbred-hybrid groups are shown for each of these genes in Additional file 7.
